# Physiological and Psychological Effects of a Forest Therapy Program on Middle-Aged Females

**DOI:** 10.3390/ijerph121214984

**Published:** 2015-12-01

**Authors:** Hiroko Ochiai, Harumi Ikei, Chorong Song, Maiko Kobayashi, Takashi Miura, Takahide Kagawa, Qing Li, Shigeyoshi Kumeda, Michiko Imai, Yoshifumi Miyazaki

**Affiliations:** 1 Department of Plastic and Reconstructive Surgery, National Hospital Organization, Tokyo Medical Center, Tokyo 152-8902, Japan; ochiroko@gmail.com; 2 Center for Environment, Health and Field Sciences, Chiba University, Chiba 277-0882, Japan; ikei0224@ffpri.affrc.go.jp (H.I.); crsong1028@chiba-u.jp (C.S.); 3 Forestry and Forest Products Research Institute, Ibaraki 305-8687, Japan; kagawa@ffpri.affrc.go.jp; 4 Department of Hygiene and Public Health, Nippon Medical School, Tokyo 113-8602, Japan; mk831111@nms.ac.jp (M.K.); qing-li@nms.ac.jp (Q.L.); 5 Agematsu Town Office Industry & Tourism Department, Nagano 399-5601, Japan; agkk5180@gmail.com; 6 Nagano Prefectural Kiso Hospital, Nagano 397-8555, Japan; kumeda@titan.ocn.ne.jp; 7 Le Verseau Inc., Tokyo 156-0051, Japan; leverseau@mvb.biglobe.ne.jp

**Keywords:** forest therapy program, middle-aged females, pulse rate, salivary cortisol, semantic differential method, Profile of Mood State

## Abstract

The natural environment is increasingly recognized as an effective counter to urban stress, and “Forest Therapy” has recently attracted attention as a relaxation and stress management activity with demonstrated clinical efficacy. The present study assessed the physiological and psychological effects of a forest therapy program on middle-aged females. Seventeen Japanese females (62.2 ± 9.4 years; mean ± standard deviation) participated in this experiment. Pulse rate, salivary cortisol level, and psychological indices were measured on the day before forest therapy and on the forest therapy day. Pulse rate and salivary cortisol were significantly lower than baseline following forest therapy, indicating that subjects were in a physiologically relaxed state. Subjects reported feeling significantly more “comfortable,” “relaxed,” and “natural” according to the semantic differential (SD) method. The Profile of Mood State (POMS) negative mood subscale score for “tension–anxiety” was significantly lower, while that for “vigor” was significantly higher following forest therapy. Our study revealed that forest therapy elicited a significant (1) decrease in pulse rate, (2) decrease in salivary cortisol levels, (3) increase in positive feelings, and (4) decrease in negative feelings. In conclusion, there are substantial physiological and psychological benefits of forest therapy on middle-aged females.

## 1. Introduction

The term “forest bathing” was proposed in Japan in 1982, and penetrated as words to express for enjoying the comfort of the forest. However, there was little information regarding “What is so psychologically comforting about the forest?” and “What specific psychological and physiological changes are taking place in a body in the forest?” The elucidation of the phenomenon rapidly advanced around the past 10 years, and it developed into the term “forest therapy” programs. Indeed, “forest therapy” is now increasingly recognized as an effective relaxation and stress management activity with demonstrated a preventive medical effect and increased healthy effect among healthy Japanese adults [1].

Several studies have shown that time spent in a forest can decrease blood pressure (BP) [2,3,4,5,6], pulse rate [2,3,4,5,6,7], sympathetic nervous activity [4,5,6,8,9,10], and cortisol levels [2,3,4,5,7,8,11,12], while increasing parasympathetic nervous activity [3,4,5,6,7,8,9,10]. Furthermore, forest stimulation decreased cerebral blood flow in the prefrontal cortex [12], and Bratman *et al.* reported that a brief nature experience decreased both self-reported rumination and neural activity in the subgenual prefrontal cortex (sgPFC) [13]. These studies suggest that accessible natural areas are a critical resource for improving mental health in our rapidly urbanizing world [13].

It was also shown that a forest therapy trip can increase human natural killer (NK) cell activity and improve immunity in both males and females, and these effects were found to last for at least 7 days [14,15,16,17]. Additionally, psychological studies have demonstrated that the negative mood was significantly lower and the positive mood was significantly higher after durations of stay in the forest [10,18].

Park *et al.* reported relaxation and stress-management effects of forest environments using several questionnaire-based metrics, in addition to improved mood [19]. In psychological tests of young adult males, forest therapy significantly increased positive feelings and reduced negative feelings in comparison with urban stimuli [2,3,4,6,8,9,10,11,12]. A majority of studies involving forest therapy experiments report the various effects in male subjects [4,8,9,10,19,20,21]; however, few reports have focused on female subjects [16].

Most field experiments on forest therapy have enrolled only healthy young adults as subjects, while those who need these benefits the most may be older adults at a higher risk of stress- and lifestyle-related diseases such as high BP, diabetes, and depression. Song *et al.* reported physiological and psychological relaxation effects on hypertensive individuals after a brief walk in the forest [20]; however, few studies have examined the effects of a standardized forest therapy program on higher-risk populations, particularly a program that can be completed within a day for convenience and broad accessibility. To address these issues, we planned experiments to measure the effects of a standardized forest therapy program on middle-aged males with high-normal BP [21] and found that systolic and diastolic BP, urinary adrenaline, and serum cortisol levels were significantly lower than baseline following the program. While this study lacked a control group, it did provide evidence that the physical and psychological benefits of a brief forest therapy program extend to middle-aged males. Here, we investigated the physiological and psychological effects of a standard forest therapy program on middle-aged females (mean age: 62 years) to allow comparison with the previously measured effects on male subjects of similar age.

## 2. Experimental Section

### 2.1. Subjects

Seventeen Japanese females ranging in age from 40 to 73 years (62.2 ± 9.4 years; mean ± standard deviation) were recruited from the Health Promotion Center in Agematsu, Nagano Prefecture. Inclusion criteria were female aged 40 years or older. Candidates who thought it may be difficult to walk in hot weather were excluded. Six subjects were on medication for hypertension, which was well controlled. All participants were free from other diseases and psychological disorder. Body mass index (BMI) [22,23] was calculated from height and weight (BMI = weight (kg) ÷ {height (m) × height (m)) and divided into a BMI ≥ 25 group and a BMI < 25 group. At 14:00 on the day before the initiation of forest therapy, the subjects gathered in a waiting room at the Health Promotion Center; they were completely informed regarding the study aims and procedures before initiating the experiment. They received a description of the experiment, and all the subjects signed an agreement to participate. After physiological inspections and questionnaires were completed, the subjects disbanded at 16:30. To control for the effects of alcohol, the subjects did not consume alcohol during the entire study period. Participants were directed to perform normal “everyday life” activities on the day before forest therapy. This study was approved by the Ethics Committee of Nagano Prefecture Kiso Hospital and the Center for Environment, Health and Field Sciences, Chiba University, Japan, on 19 August 2013 and performed according to the Declaration of Helsinki [24].

### 2.2. Experimental Sites

The forest therapy phase was conducted in Akasawa Shizen Kyuyourin (Akasawa Natural Recreation Forest), Agematsu, Nagano Prefecture (situated in central Japan) on 30 August 2014. The distance from the health promotion center to the forest was 14.6 km, and it took 42 min to drive by car. The weather was cloudy on the forest therapy day, with a mean temperature of 21.5 °C (18.2 °C–27.5 °C) and humidity of 81% (49%–96%).

### 2.3. Physiological Indices

Both systolic and diastolic BP levels and pulse rate readings were obtained from the right arm using a portable digital sphygmomanometer (HEM-1020, Omron, Kyoto, Japan).These procedures were performed between 15:09 and 15:22 on the day before forest therapy and between 14:44 and 14:56 after forest therapy to control for circadian effects.

Salivary cortisol, which shows a reliable increase under stress, was measured as an index of endocrine activity. Saliva samples were collected using a saliva collection aid (No.61/524,096; SalivaBio LLC, California, USA) between 15:28 and 15:35 on the day before forest therapy and between 14:57 and 15:05 after forest therapy. Saliva samples collected at the field site were immediately placed in a freezer and sent to a laboratory (MACROPHI Inc, Takamatsu, Japan) for analysis.

### 2.4. Psychological Indices

The semantic differential (SD) method and a short form of the Profile of Mood State (POMS) were used to evaluate psychological responses to forest therapy. These questionnaires were completed by subjects between 15:00 and 15:20 on the day before forest therapy and between 14:44 and 14:56 after forest therapy. The SD method uses three pairs of adjectives anchoring seven-point scales: “comfortable to uncomfortable,” “relaxed to awakening,” and “natural to artificial” [25]. The short form of POMS was used to decrease the burden on the subjects [26]. We assessed three subscales: “tension–anxiety,” “fatigue,” and “vigor.”

### 2.5. Experimental Design

**Figure 1:**
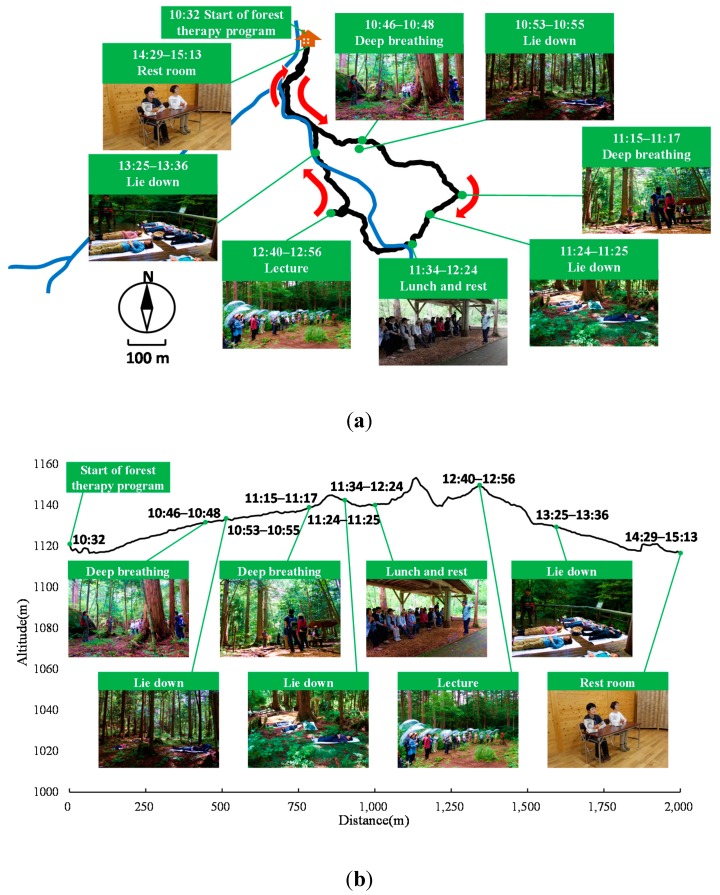
Images showing the various activities of the forest therapy program with location map. (**a**): plane map, (**b**): altitude map.

The subjects spent the previous night in their respective homes. On the morning of the forest therapy day, the subjects gathered in the same meeting room at 9:00 and participated in the forest therapy program as a group with a guide. They were not permitted to carry cell phones. The program consisted of multiple timed activities over 4 h and 41min (Table 1) led by a guide. The subjects walked around their assigned area and then sat and lay on their backs in the forest on waterproof sheets laid on the ground during rest breaks. The guide put on measuring equipment with a map-caching offline GPS application (Geographica, Japan) and accompanied the subjects in the forest (Figure 1a,b).

**Table 1:** Time schedules and calorie consumption during various activities of the forest therapy program.

Time	Event	Calorie Consumption (Kcal/min)
10:32–10:45	Stroll (Forest )	1.21
10:46–10:48	Deep breathing (Forest)	0
10:49–10:52	Stroll (Forest)	0.15
10:53–10:55	Lie down (Forest)	0
10:56–11:14	Stroll (Forest)	0.65
11:15–11:17	Deep breathing (Forest)	0.10
11:18–11:23	Stroll (Forest)	0.48
11:24–11:25	Lie down (Forest)	0.06
11:26–11:33	Stroll (Forest)	0.52
11:34–12:24	Lunch and rest (Resting room)	0.04
12:25–12:39	Stroll (Forest)	0.92
12:40–12:56	Lecture (Forest)	0.08
12:57–13:09	Stroll (Forest )	0.66
13:10–13:24	Rest (Forest)	0.01
13:25–13:36	Lie down & abdominal breathing (Forest)	0.00
13:37–13:59	Chat (Forest)	0.01
14:00–14:28	Stroll (Forest)	0.76
14:29–15:13	Rest (Resting room)	0.02

Energy expenditure was assessed for each activity using Lifecorder GS4 (Suzuken Co., Ltd., Chiba, Japan). Tobacco and all drinks (except mineral water) were prohibited during forest therapy. The subjects ate the same lunch made from local ingredients at the same time (11:34–12:24). After the subjects completed the program, they returned to a waiting room for post-treatment measurements and to complete the questionnaires. These results were then compared with those obtained on the previous day.

We aimed to compare the physiological and psychological effects of forest therapy with everyday life activities on a normal day. Physiological and psychological inspections were performed at approximately the same time on the day before and on the day of the therapy.

### 2.6. Statistical Analysis

We used paired *t*-tests to compare physiological indices and the Wilcoxon signed-rank test to compare psychological test results obtained before and immediately after forest therapy. All statistical analyses were performed using SPSS 20.0 (IBM Corp., Armonk, NY, USA). Data are expressed as the mean ± standard error (mean ± SE). For all tests, *p* < 0.05 (one sided) was considered statistically significant.

## 3. Results

**Figure 2:**
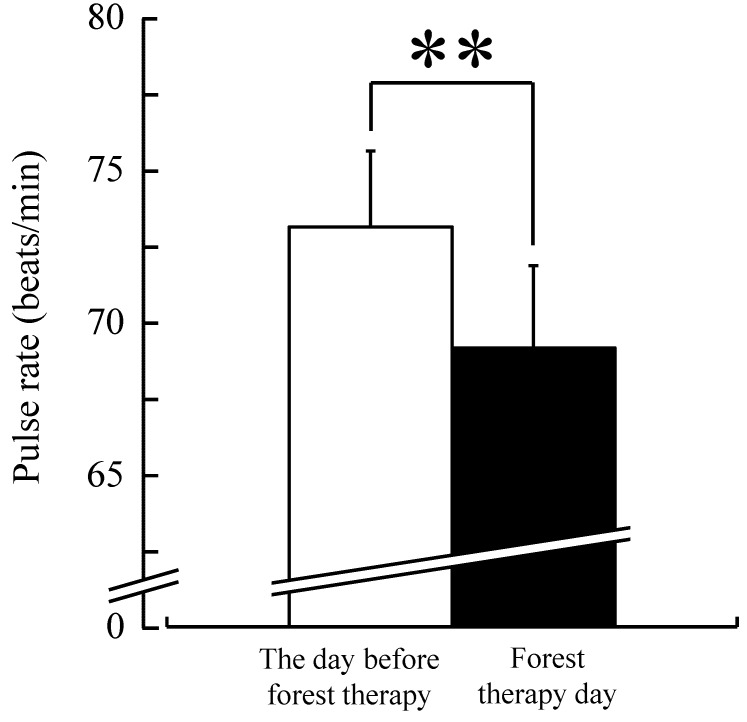
Effect of forest therapy on pulse rate of middle-aged females. N = 17, mean ± standard error. ** *p* < 0.01, paired *t*-test.

**Figure 3:**
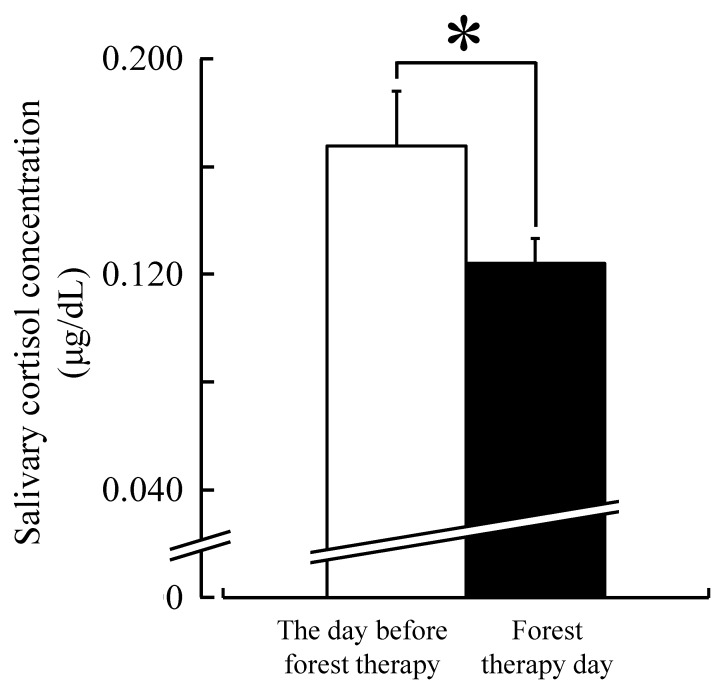
Effect of forest therapy on salivary cortisol level. N = 17, mean ± standard error. * *p* < 0.05, paired *t*-test.

Pulse rate was significantly lower after forest therapy than on the day before forest therapy (baseline) in middle-aged females (69.1 ± 2.7 *vs.* 73.1 ± 2.5 beats/min; t(16) = 4.67, *p* < 0.01 by paired *t*-test) (Figure 2). Similarly, salivary cortisol levels were significantly lower after forest therapy than on the day before forest therapy (0.124 ± 0.009 *vs.* 0.168 ± 0. 020 μg/dL; t(16) = 2.63, *p* < 0.05 by paired *t*-test) (Figure 3).

The total energy expenditure during forest therapy was compared between subjects with BMI ≥ 25 (N = 4) and those with BMI < 25 (N = 13). A marginally significant difference was observed between groups, with 24% greater expenditure in the BMI ≥ 25 group compared with the BMI < 25 group (0.88 ± 0.08 *vs.* 0.71 ± 0.06 kcal/min; t(15) = 1.88, *p* < 0.10 by unpaired *t*-test). The mean salivary cortisol level was reduced in the BMI < 25 group after forest therapy (0.186 ± 0.024 *vs.* 0.123 ± 0.012μg/dL; t(12) = 3.19, *p* < 0.01 by paired *t*-test), but it actually increased slightly in the BMI ≥ 25 group (0.109 ± 0.013 *vs.* 0.128 ± 0.014μg/dL; t(3) = 4.01, *p* < 0.05 by paired *t*-test).

**Figure 4:**
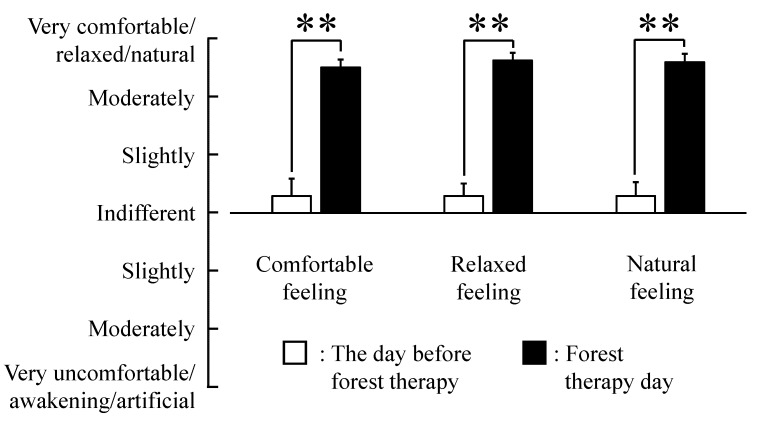
Semantic differential (SD) method scores for the day before forest therapy and immediately after forest therapy, showing changes in the subjective feelings “comfortable,” “relaxed,” and “natural”. N = 17, mean ± standard error. ** *p* < 0.01, Wilcoxon signed-rank test.

**Figure 5:**
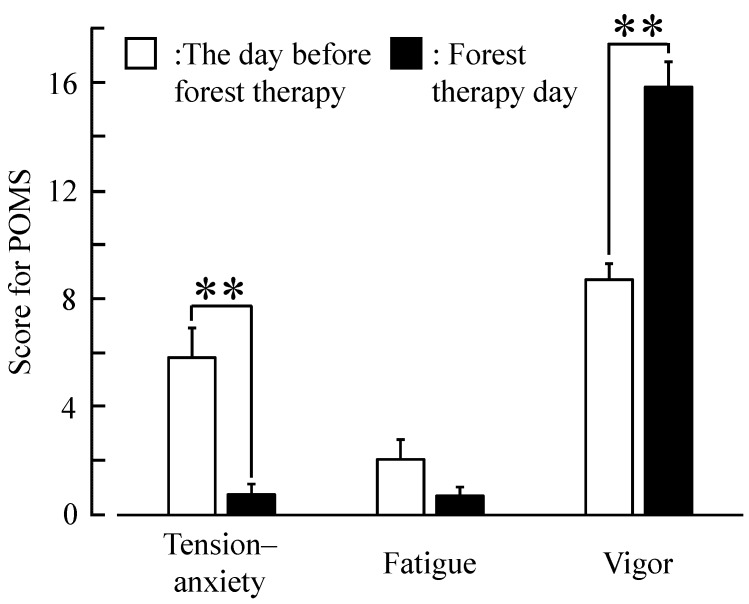
Lower negative and higher positive subjective Profile of Mood State (POMS) subscores after forest therapy than on the day before forest therapy. N = 17, mean ± standard error. ** *p* < 0.01, Wilcoxon signed-rank test.

Significantly higher SD scores were observed for the adjectives “comfortable” (*p* < 0.01), “relaxed” (*p* < 0.01), and “natural” (*p* < 0.01) after forest therapy than on the day before forest therapy (Figure 4). Finally, a significant elevation of mood was detected on POMS (Figure 5), with scores for the negative subscale “tension–anxiety” being significantly lower (*p* < 0.01) and those for the positive subscale “vigor” (*p* < 0.01) being significantly higher after forest therapy.

## 4. Discussion

The present study assessed the physiological and psychological benefits of forest therapy on middle-aged Japanese females. The mean pulse rate was significantly lower after walking in a forest environment than on the day before forest therapy. Because the pulse rate is a basic index of autonomic nervous system activation, the drop in pulse rate indicates a state of relaxation in middle-aged females, consistent with the past reports that examined physiological responses to a natural environment in young adults [2,3,4,5,6,7]. Thus, we concluded that this benefit of physiological relaxation extends to middle-aged females.

Sympathetic activity can be determined by measuring the levels of urinary adrenaline and/or noradrenaline [27], and many previous studies have shown that reducing stress decreases sympathetic activity, as measured by systemic cortisol levels [28]. The concentration of cortisol was the highest immediately after waking up and decreases and stabilizes in the afternoon. We used saliva samples for measuring cortisol levels as this method is easily manageable in a field setting and is non-invasive. Furthermore, salivary cortisol provides a reliable prediction of total and calculated free serum cortisol levels [29]. It has been reported that the normal level of salivary cortisol is 0.07–0.73 μg/dL [30]. Many previous studies have shown that lowered stress levels result in lower cortisol levels [2,3,4,5,7,8,11,12]; therefore, we conclude that forest therapy also reduces stress in middle-aged females.

For several decades, BMI (kg/m^2^) has been used to diagnose obesity in clinical practice and obesity research and to structure programs and goals for weight loss interventions [31]. BMI is sometimes used to estimate total body fat and determine whether a person has a healthy weight. While BMI may not always provide an accurate estimate of excess body fat, BMI ≥ 25 is linked to increased risk of diseases such as heart disease and some cancers. In the present study, the salivary cortisol level was reduced only in subjects with BMI < 25. Note, however, that these measures are derived for a single forest therapy session, which may have been more stressful on the heavier subjects. Therefore, for middle-aged females with high BMI, a sustained regular program may be necessary for the anti-stress benefits to emerge.

The baseline salivary cortisol level was 1.7-fold higher in subjects with BMI < 25 than in those with BMI ≥ 25, but this difference disappeared after forest therapy. Song *et al.* reported that subjects with high initial BP showed a decrease, while those with low initial values showed an increase after walking in a forest area [32]. These results suggest a physiological adjustment effect in the forest environment, which may also account for the normalization of cortisol levels among participants with different BMI. However, no report has studied this adjustment effect for cortisol levels in subjects matched for baseline BMI; therefore, additional studies are necessary.

According to the SD questionnaires, middle-aged females felt more “comfortable,” “natural,” and “relaxed” after forest therapy. In addition, the negative emotion “tension–anxiety” was reduced and the positive feeling of “vigor” was higher after forest therapy according to the short form of POMS. Similarly, middle-aged males reported feeling significantly more “natural” and “relaxed” after walking in a forest [21]. While “tension–anxiety” was significantly lower after forest therapy in middle-aged males as well, in contrast to middle-aged females, they reported no significant change in “vigor” [21]. Neither group reported changes in “fatigue,” although measurement immediately after the forest walk may have contributed to temporary fatigue. Nonetheless, these findings indicate that a single forest therapy session has psychological benefits for both middle-aged women and men.

Although many factors can affect the general condition of menstruating females, little is known about differences in the relationship between physiological and subjective stress responses at various phases of the menstrual cycle [33]. Watanabe *et al.* reported that no significant differences in salivary cortisol levels were observed during any phase of the menstrual cycle [34]. Additionally, the mean age of this study sample was 62 years. However, because the mean age for menopause in Japanese women is approximately 50 years, we did not consider the influence of the menstrual cycle in this experiment. Menopausal disorders are a frequent problem in middle-aged females. Many of these problems may stem from disruption of the intricate links between estrogen metabolism and the autonomic nervous system. Many women gain weight because of the decrease in estrogen and basal metabolism, while autonomic changes may lead to tachycardia and mental health effects. Normal aging influences various indices, and the parasympathetic tone is generally higher in women than men, as evidenced by heart rate variability (HRV) measurements [35]. It has been reported that physical activity facilitates improved HRV stability in older women and that the quantity of exercise training necessary for such an improvement is relatively modest [36].

“Forest therapy” is increasingly recognized not only as a convenient exercise but also as a relaxation and stress management activity with demonstrated clinical benefits [1]. Moreover, we can control the energy expenditure by choosing the appropriate course terrain, distance, and walking speed and by including regular rest and relaxation sessions, such as sitting, lying, and deep breathing. Forest therapy could be an effective and convenient method for the improvement of menopausal symptoms such as autonomic imbalance, stiff shoulder, knee pain, constipation, shortness of breath, and depression. Furthermore, as a group activity, forest therapy is an opportunity to spend time enjoying the natural environment with friends and family.

The present study provides evidence for physiological and psychological benefits of forest therapy in middle-aged females. Limitations of the present study include the lack of a control group performing similar activities in an urban environment. An ideal experimental design would include a comparison of the effects of forest therapy using the same parameters/environmental stimuli, but instead conducting the comparison (control group) in an urban area setting. However, this would be difficult to implement in practice because it involves activities such as “lying down” in an urban area. So the control experiments of the same activities completed indoors are thought to be necessary in future. Interestingly, differences in effect were observed with varying BMI. However, the limited number of subjects in this study decreased the significance of the analysis. Future studies should include a larger number of subjects. Furthermore, forest therapy has not yet been shown to actually reduce the risk of disease independent of the general effects of exercise. It is now necessary to design experiments that test whether forest therapy can reduce disease risk in vulnerable populations through these demonstrated physiological and psychological benefits.

## 5. Conclusions

Our study revealed that forest therapy elicited a significant (1) decrease in pulse rate, (2) decrease in salivary cortisol levels, (3) increase in “comfortable,” “natural,” and “relaxed” feelings as assessed by the modified SD method, (4) decrease in the POMS negative subscale “tension–anxiety,” and (5) increase in feelings of “vigor” in middle-aged females. In conclusion, walking in a forest according to a standard “forest therapy” program induced physiological and psychological relaxation. These results clarified the physiological effects of the forest therapy program and suggested a possibility of clinical use.
